# Elutriated lymphocytes for manufacturing chimeric antigen receptor T cells

**DOI:** 10.1186/s12967-017-1160-5

**Published:** 2017-03-16

**Authors:** David F. Stroncek, Daniel W. Lee, Jiaqiang Ren, Marianna Sabatino, Steven Highfill, Hanh Khuu, Nirali N. Shah, Rosandra N. Kaplan, Terry J. Fry, Crystal L. Mackall

**Affiliations:** 1 Cell Processing Section, Department of Transfusion Medicine, NIH Clinical Center, NIH, 10 Center Drive-MSC-1184, Building 10, Room 3C720, Bethesda, MD 20892-1184 USA; 2 Pediatric Oncology Branch, Center for Cancer Research, National Cancer Center, NIH, Bethesda, USA; 3 Division of Pediatric Hematology/Oncology, Department of Pediatrics, University of Virginia, Charlottesville, USA; 4 Parker Institute for Cancer Immunotherapy, Stanford University, Stanford, USA

**Keywords:** Chimeric antigen receptor T cells, Cancer immunotherapy, Cellular therapy, T cells, Elutriation, Myeloid derived suppressor cells, Peripheral blood mononuclear cells

## Abstract

**Background:**

Clinical trials of Chimeric Antigen Receptor (CAR) T cells manufactured from autologous peripheral blood mononuclear cell (PBMC) concentrates for the treatment of hematologic malignancies have been promising, but CAR T cell yields have been variable. This variability is due in part to the contamination of the PBMC concentrates with monocytes and granulocytes.

**Methods:**

Counter-flow elutriation allows for the closed system separation of lymphocytes from monocytes and granulocytes. We investigated the use of PBMC concentrates enriched for lymphocytes using elutriation for manufacturing 8 CD19- and 5 GD2-CAR T cell products.

**Results:**

When compared to PBMC concentrates, lymphocyte-enriched elutriation fractions contained greater proportions of CD3+ and CD56+ cells and reduced proportions of CD14+ and CD15+ cells. All 13 CAR T cell products manufactured using the elutriated lymphocytes yielded sufficient quantities of transduced CAR T cells to meet clinical dose criteria. The GD2-CAR T cell products contained significantly more T cells and transduced T cells than the CD19-CAR T cell products. A comparison of the yields of CAR T cells produced from elutriated lymphocytes with the yields of CAR T cells previous produced from cells isolated from PBMC concentrates by anti-CD3/CD28 bead selection or by anti-CD3/CD28 bead selection plus plastic adherence found that greater quantities of GD2-CAR T cells were produced from elutriated lymphocytes, but not CD19-CAR T cells.

**Conclusions:**

Enrichment of PBMC concentrates for lymphocytes using elutriation increased the quantity of GD2-CAR T cells produced. These results provide further evidence that CAR T cell expansion is inhibited by monocytes and granulocytes.

## Background

Early phase clinic trials of T cells genetically engineered to express chimeric antigen receptors (CAR) have been encouraging. CD19-CAR T cells have been used successfully in a number of clinical trials to treat non-Hodgkin lymphoma and acute lymphocytic leukemia (ALL) [1–8]. Preliminary studies of B cell maturation antigen (BCMA)-CAR T cells to treat multiple myeloma have also been promising [9].

Most CAR T cell manufacturing protocols initate cell production with autologous T cells collected by apheresis using a blood cell separator which separates lymphocytes from plasma, platelets, red blood cells (RBCs) and granulocytes. However, the lymphocyte-rich peripheral blood mononuclear cell (PBMC) concentrates collected by apheresis are also enriched for monocytes and contain variable quantities of RBCs, platelets and granulocytes. The quantities of these contaminating cells are dependent on the type of blood cell separator and how the blood cell separator is operated. The composition of the PBMC concentrates are also dependent on the type of tumor (solid vs. liquid), and the patient’s blood counts at the time of collection [10]. While the quantities of these contaminating RBCs, platelets and granulocytes cells can be minimized with highly trained users of the cell separator instrument, they cannot be completely eliminated. Consequently, prior to beginning the CAR T cell manufacturing process the PBMC concentrates are generally enriched for lymphocytes or CD3+ cells in the cell processing laboratory.

Our center initially manufactured CD19- and GD2-CAR T cells using autologous PBMC concentrates enriched for T cells by magnetic selection with the anti-CD3/CD28 beads. These same anti-CD3/CD28 beads were also used to stimulate T cell expansion. While the method was, in general, effective, we found that the quantities of GD2-CAR T cells produced were less than the quantities of CD19-CAR T cells produced [11]. In addition, CAR T cells from some patients failed to expand to sufficient levels to meet patient treatment dose criteria. Upon further investigation, we discovered that the presence of large quantities of monocytes or granulocytes in some PBMC concentrates was associated with poor in vitro expansion of CAR T cells [11]. We modified the T cell enrichment method to include a plastic adherence step to deplete PBMC concentrates of monocytes prior to the anti-CD3/CD28 bead enrichment step. This modified T cell enrichment process improved T cell expansion, but it was not completely effective at removing contaminating monocytes and granulocytes and did not completely eliminate manufacturing failures [11].

We hypothesized that more rigorous enrichment of the starting material for lymphocytes would improve the yield of transduced T cells and reduce the incidence of manufacturing failures. A semi-automated counter-flow elutriation instrument is available for enriching PBMC concentrates for monocytes and lymphocytes which makes use of a sterile single use disposable kit [12].

We modified our CAR T cell manufacturing process to include elutriation for the enrichment of PMBC concentrates for lymphocytes rather than anti-CD3/CD28 bead selection or anti-CD3/CD28 bead selection plus plasitic adherence. We report the results of manufacturing CD19- and GD2-CAR T cells using lymphocytes collected by apheresis and enriched by elutriation as starting material. We also compared CD19- and GD2-CAR T cells manufactured from elutriated lymphocytes with those that we previously manufactured from PBMC concentrates that were enriched for lymphocytes with anti-CD3/CD28 bead selection or bead selection plus plastic adherence [11].

## Methods

### Study participants

Patients in this study were enrolled in an open-label phase 1 dose-escalation study of CD19-CAR T cells in children and young adults with ALL or non-Hodgkin lymphoma, NCT01593696, or an open-label phase 1 dose-escalation study of GD2-CAR T cell in children and young adults with GD2 expressing osteosarcoma or neuroblastoma, NCT02107963. Clinical results of the first 21 of the patients receiving CD19-CAR T cell therapy have previously been reported [1]. The results of CD19-CAR T cell manufacturing from the first 43 patients and GD2-CAR T cells from the first 11 patients have previous been reported [11]. This study reports the results of manufacturing CD19-CAR T cells from 8 additional patients and GD2-CAR T cells from 5 additional patients. Among the 8 patients treated with CD19-CAR T cells 7 had ALL and one had diffuse large B cell lymphoma (DLBCL). All 5 patients treated with GD2-CAR T cells all had osteosarcoma. All 8 patients receiving CD19-CAR T cells were given a dose of 1 × 10^6^ cells/kg and 2 patients receiving GD2-CAR T cells were given a dose of 3 × 10^6^ CAR T cells/kg and 3 patients were given a dose of 1 × 10^7^ CAR T cells/kg. All subjects were enrolled in protocols approved by the National Cancer Institute (NCI) Institutional Review Board and inform consent was obtained.

We also compared the results of manufacturing CD19- and GD2-CAR T cells in this study with the previously reported results of manufacturing CD19- and GD2-CAR T cells using PBMC concentrates enriched for T cells by selection with anti-CD3/CD28 beads with and without plastic adherence [11].

### Manufacturing CAR T cells

Peripheral blood mononuclear cells concentrates were collected using a blood cell separator (Cobe Spectra, Terumo BCT, Lakewood, CO, USA) and 10–15 L of blood was processed. CD19-CAR T cells were manufactured from PBMC concentrates using a modification of the method we previously described [11, 13]. When PBMCs were enriched for lymphocytes by anti-CD3/CD28 bead selection, on day 0, a fresh or cryopreserved PBMC concentrate containing 600 × 10^6^ CD3+ cells were enriched for CD3+ cells using anti-CD3/CD28 antibodies bound to paramagnetic beads (Dynabeads ClinExVivo CD3/CD28, Invitrogen, Camarillo, CA) at a ratio of 3:1 (beads:cells). The cells and beads were co-incubated for 2 h at room temperature and CD3+ cell enrichment was performed using Dynal ClinExVIVO MPC magnet (Invitrogen, Camarillo, CA). A total of 100 × 10^6^ cells in the CD3+ fraction were resuspended at a concentration of 1 × 10^6^ cells/mL in PermaLife bags (OriGen Biomedical, Austin, TX) at 37 °C in 5% CO_2_ in AIM V medium (Gibco, Grand Island, NY), supplemented with 5% heat-inactivated human AB Serum (Valley Biomedical, Winchester, VA), 1% Gluta-Max (Gibco, Grand Island, NY), 40 IU/mL IL-2 (Novartis Vaccines and Diagnostics, Inc. Emeryville, CA).

For manufacturing CD19-CAR T cells, the lymphocyte enriched cells were transduced twice with clinical grade MSGV-FMC63-28Z recombinant retroviral vector supernatant, once on day 2 and once on day 3, in retronectin-coated bags. The cells were maintained in culture for 7–9 days. The cell concentration was maintained at 0.4 × 10^6^ cells/mL by adding fresh media every other day. On the day of harvest the anti-CD3/CD28 paramagnetic beads were removed using the Dynal ClinExVIVO MPC magnet (Invitrogen, Camarillo, CA), washed, concentrated and quality control assessment was performed.

For manufacturing GD2-CAR T cells, the same lymphocyte enrichment processes was used and a similar process was used to transduce and expand the cells. Cells were transduced once on day 2 with anti-GD2.28.z.OX40.ICD9 retroviral vector supernatant and were harvested after 10 or 11 days in culture.

When PBMC concentrates were enriched for lymphocytes by anti-CD3/CD28 bead selection plus plastic adherence the PBMC concentrates were incubated with anti-CD3/CD28 beads for 2 h in T flasks rather than in bags. At the end of the 2 h incubation period the non-adherent cells were collected and the cells were processed as described above.

When the PBMC concentrates were enriched for lymphocytes by elutriation they were subject to elutriation using a semi-automatic counter-flow elutriation instrument (Elutra Cell Separation System, version 1.1, Terumo BCT) using a user defined profile which collects cells in 5 fractions. The chamber rotation speed was maintained at 2400 RPMs for fractions 1 through 4 and the media flow rate was maintained at 60 mL/min for fraction 1, 120 mL/min for fraction 2, 122 mL/min for fraction 3 and 124 mL/min for fraction 4. Fraction 5 was the cells remaining in the chamber and they were collected with the rotor turned off. Lymphocytes were found in fractions 1 and 2 and monocytes and granulocytes were in fraction 5. Fraction 1 and 2 also were enriched with platelets and RBCs [12].

The lymphocyte fraction was depleted of RBCs by lysis. The cells were pelleted by centrifugation, incubated for 7–10 min with ACK Lysing Buffer (Lonza, Walkersville, MD) and then washed and resuspended in 0.9% saline (B.Braun Medical Inc., Irvine, CA) with 0.3% trisodium citrate (TriCitrasol anticoagulant sodium citrate concentrate 46.7%, Citra Labs, Braintree, MA).

### Cell counts and flow cytometry

Blood counts were measured using automated hematology analyzer (Cell-Dyn 3700). Flow cytometry was performed with a FACSCanto II (BeckinDickinson) using CD3, CD4, CD8, CD14, CD15, CD19, CD45 and CD56 antibodies (BD Biosciences, San Jose, CA). The expression of CD19-CAR and GD2-CAR was assessed by flow cytometry with anti-idiotype antibodies.

### Statistically analysis

The values shown are mean ± 1 standard deviation unless otherwise indicated. Groups were compared using *t*-tests (Microsoft Excel, Microsoft Inc., Redmond, WA).

## Results

### Composition of the PBMC concentrates and elutriated lymphocytes

We manufactured 8 CD19 CAR T cell products and 5 GD2-CAR T cell products using autologous PBMC concentrates collected by apheresis and enriched for lymphocytes by elutriation. The PBMC concentrates, as expected, contained significant quantities of monocytes and neutrophils in addition to the desired lymphocytes (Table 1).

**Table 1:** Proportion of leukocytes in autologous PBMC concentrates collected for CD19- and GD2-CAR T cell manufacturing

Cell type	CAR T cell type			
	n	CD19 (n = 8)	GD2 (n = 5)	All (n = 13)
Neutrophils	(%)	11.0 ± 18.2 (1–58)^a^	5.2 ± 3.6 (1–10)	8.8 ± 14.4 (1–58)
Lymphocytes	(%)	59.1 ± 24.1 (7–84)	65.8 ± 6.8 (58–78)	61.7 ± 24.7 (7–89)
Monocytes	(%)	27.1 ± 12.3 (8–45)	26.4 ± 6.2 (19–38)	26.8 ± 12.2 (8–45)

^a^Values represent the mean ± SD and range

To assess the effectiveness of elutriation for lymphocyte enrichment the PBMC concentrates and the elutriated lymphocyte fractions were analyzed by flow cytometry with anti-CD3, anti-CD19, anti-CD14, anti-CD15 and anti-CD56 for T cells, B cells, monocytes, neutrophils and NK cells, respectively (Table 2). Among all of the procedures analyzed the elutriated lymphocytes were enriched for CD3+ cells and CD56+ cells and had reduced levels of CD14+ and CD15+ cells. There was no difference in the proportion of CD19+ cells in the PBMC concentrates and lymphocytes fraction. The elutriated lymphocytes from patients enrolled in both the CD19- and GD2-CAR T cell protocols contained a greater proportion of CD3+ and less CD14+ cells than the PBMC concentrates. The lymphocyte fraction from CD19-CAR T patients was also enriched for CD56+ cells.

**Table 2:** Comparison of the composition of PBMC concentrates and the elutriated lymphocyte fraction used to manufacture CD19- and GD2-CAR T cells

Cell type	CAR T cell type								
	CD19			GD2			All		
	n	PBMC	Elutriated lymphs	n	PBMC	Elutriated Lymphs	n	PBMC	Elutriated lymphs
CD3+	8	49.0 ± 23.1	62.1 ± 24.4^a^	5	42.2 ± 17.0	58.0 ± 23.0^a^	13	49.3 ± 22.5	64.6 ± 25.5^a^
CD14+	8	17.2 ± 11.4	3.5 ± 1.7^a^	4	20.9 ± 9.7	3.9 ± 3.2^a^	12	19.9 ± 13.7	3.6 ± 4.8^a^
CD15+	8	8.2 ± 15.2	4.1 ± 10.6	4	3.9 ± 2.8	0.8 ± 1.4	12	6.9 ± 12.0	2.9 ± 8.2^a^
CD19+	8	13.8 ± 16.9	13.4 ± 16.4	2	10.4 ± 6.7	14.5 ± 9.2	10	14.0 ± 14.7	11.9 ± 8.6
CD56+	8	7.4 ± 4.9	12.6 ± 8.5^a^	2	4.8 ± 2.8	6.8 ± 3.8	10	7.2 ± 5.0	11.9 ± 8.6^a^

^a^p < 0.05 Paired T-tests of pre- and post-elutriation samples

Values represent the mean ± SD

### Manufacturing CD19- and GD2-CAR T cells using elutriated lymphocytes

All 13 CAR T cell manufacturing procedures yielded sufficient quantities of T cells to meet the dose criteria (1 × 10^6^/kg for CD19-CAR T cells and 3 × 10^6^ or 1 × 10^7^ cells/kg for GD2-CAR T cells). The 13 CAR T cell products contained 2166 ± 1113 × 10^6^ CD3+ cells and 1064 ± 877 × 10^6^ transduced CD3+ T cells. The GD2-CAR T cell final products contained more total nucleated cells (TNC), CD3+ cells and transduced CD3+ cells than the CD19-CAR T cell final products (Table 3). There was no difference among CD19- and GD2-CAR T cell final products in the proportion of CD3+ cells or transduced CD3+ cells (Table 3). The proportion of CD3+ cells that expressed CD4 or CD8 was also similar among CD19- and GD2-CAR T cell final products (Table 3). The proportion of CD19+ cells was also measured in the CD19-CAR T cell final products and very few were detected, 0.1 ± 0.1%, range = 0.0–0.2%.

**Table 3:** Quantity of total nucleated cells, CD3+ cells and transduced CD3+ cells in the final CAR T cell products and CD4+ and CD8+ cell content of the final products

Cell type	CAR T cell type					
	CD19 (n = 8)		GD2 (n = 5)		All (n = 13)	
	Mean ± SD	Range	Mean ± SD	Range	Mean ± SD	Range
TNC (×10^6^)	981 ± 235	668–1440	4384 ± 1194^a^	2250–6610	2289 ± 1865	668–6160
CD3+ cells (×10^6^)	946 ± 273	500–1434	4119 ± 934^a^	2499–5119	2166 ± 1133	500–5119
Transduced CD3+ cells (×10^6^)	517 ± 223	150–763	1940 ± 775^a^	837–3018	1064 ± 877	150–3018
CD3+ cells (%)	95.4 ± 8.6	74.9–99.9	95.1 ± 6.0	83.1–98.7	95.3 ± 25.5	74.9–99.9
Transduced CD3+ cells (%)	52.8 ± 15.0	30.0–69.3	45.7 ± 12.0	33.5–63.7	50.1 ± 21.3	30.0–69.3
CD4+ cells (%)	50.6 ± 14.8	24.9–72.3	38.1 ± 17.2	14.1–57.1	45.8 ± 19.7	14.1–72.3
CD8+ cells (%)	42.4 ± 16.5	18.3–65.7	53.3 ± 15.2	35.3–74.1	45.6 ± 19.6	18.3–74.1

^a^p < 0.05 for comparison of CD19- and GD2-CAR T cells

### Comparison of CAR T cells manufactured using lymphocytes enriched by anti-CD3/CD28 bead selection with and without plastic adherence and elutriation

**Fig. 1:**
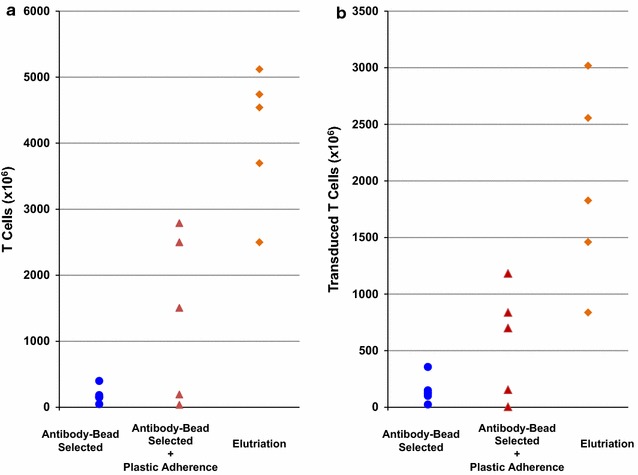
Quantity of T cells and transduced T cells in GD2-CAR T cell products. GD2-CAR T cells were manufactured over 10 or 11 days using autologous PBMC concentrates enriched for lymphocytes using one of 3 methods: anti-CD3/CD28 bead selection (n = 6, *circles*), anti-CD3/CD28 bead selection plus plastic adherence (n = 5, *triangle*) and elutriation (n = 5, *diamonds*). The quantity of CD3 + cells in the final product is shown in **a** and transduced CD3+ cells in **b**

**Fig. 2:**
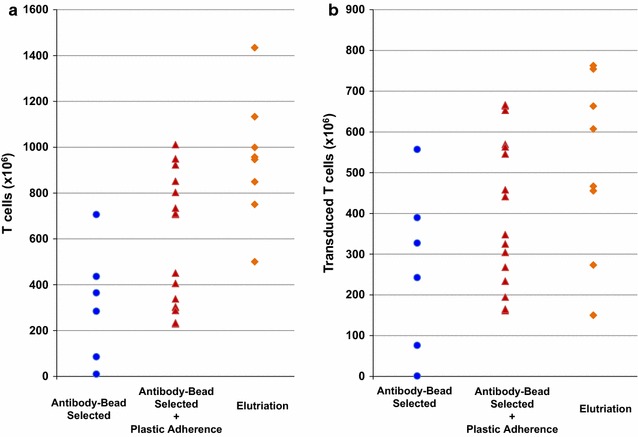
Quantity of T cells and transduced T cells in CD19-CAR T cell products. CD19-CAR T cells were manufactured over 7–9 days using autologous PBMC concentrates enriched for lymphocytes using one of 3 methods: anti-CD3/CD28 bead selection (n = 6, *circles*), anti-CD3/CD28 bead selection plus plastic adherence (n = 16, *triangle*) and elutriation (n = 8, *diamonds*). The quantity of CD3+ cells in the final product is shown in **a** and transduced CD3+ cells in **b**

We compared the yields of CAR T cells produced from PBMC concentrates enriched for lymphocytes by elutriation in this study and those produced from PBMC concentrates enriched for lymphocytes by anti-CD3/CD28 beads with and without plasitic adherence in a previous study [11]. For GD2-CAR T cell products, greater quantities of T cells were produced from elutriated lymphocytes (4119 ± 934 × 10^6^) than from anti-CD3/CD8 bead enriched (183 ± 106 × 10^6^; *p* = 6.73 × 10^−6^) and anti-CD3/CD28 bead plus adherence enriched cells (1404 ± 1136 × 10^6^; *p* = 6.14 × 10^−3^) (Fig. 1a) and greater quantities of CD3+ transduced GD2-CAR T cells were produced from elutriated lymphocytes (1940 ± 775 × 10^6^) than from anti-CD3/CD28 bead enriched (147 ± 102 × 10^6^; *p* = 6.68 × 10^−4^) and anti-CD3/CD28 bead plus adherence enriched cells (576 ± 437 × 10^6^; *p* = 0.015) (Fig. 1b). For CD19-CAR T cells, greater quantities of T cells were produced from elutriated lymphocytes (946 ± 273 × 10^6^) than from anti-CD3/CD28 bead enriched (305 ± 280 × 10^6^; *p* = 8.34 × 10^−4^) and bead plus adherence enriched cells (577 ± 284 × 10^6^; *p* = 6.04 × 10^−3^) (Fig. 2a), but the quantities of transduced T cells were no greater from elutriation enriched lymphocytes (517 ± 223 × 10^6^) than from anti-CD3/CD28 bead enriched (253 ± 227 × 10^6^; *p* = 0.052) or beads selection plus adherence enriched cells (410 ± 183 × 10^6^; *p* = 0.223) (Fig. 2b).

There was no difference in transduction efficiencies of T cells among the GD2-CAR T cells manufactured using PBMC concentrates enriched by elutriation and bead selection plus adherence (45.7 ± 12.0 versus 42.6 ± 22.5%; *p* = 0.81); both were transduced once. GD2-CAR T cells manufactured with cells enriched by anti-CD3/CD28 bead selection alone were transduced twice and the transduction efficiency was greater (75.1 ± 14.6%) than those manufactured with cells enriched by elutriation (*p* = 9.70 × 10^−3^) and beads plus adherence (*p* = 0.0283). All CD19-CAR T cell products were transduced twice but the transduction efficiencies were less for CD19-CAR T cells manufactured using elutriation enriched cells than anti-CD3/CD28 bead plus adherence enriched cells (52.8 ± 15.0 versus 72.7 ± 12.1%; *p* = 1.9 × 10^−3^) but not anti-CD3/CD28 bead enriched cells (76.4 ± 26.6%; *p* = 0.055).

We also compared the CD4 and CD8 composition of CAR T cells manufactured from lymphocytes enriched using the 3 different methods and found no difference in the proportion of CD3+ cells that expressed CD4 and CD8 among CAR T cells manufactured from PBMCs enriched for lymphocytes or T cells using the 3 different methods (Table 4).

**Table 4:** Comparison of the portion of CD4+ and CD8+ cells in CAR T cells manufactured from PBMC concentrates enriched for lymphocytes using 3 different methods

Lymphocyte enrichment method	CAR T cell type					
	CD19			GD2		
	n	CD4	CD8	n	CD4	CD8
Anti-CD3/CD28 beads	6	54.5 ± 26.1	35.0 ± 25.1	6	45.8 ± 12.3	48.4 ± 14.7
Anti-CD3/CD28 beads + plastic adherence	16	57.3 ± 15.7	38.4 ± 14.8	5	48.0 ± 48.7	40.0 ± 10.1
Elutriation	8	50.6 ± 14.8	42.4 ± 16.5	5	38.1 ± 17.1	53.2 ± 15.1

## Discussion

We investigated manufacturing CAR T cells using autologous PBMC concentrates enriched for lymphocytes using counter flow elutriation. The number of clinical products analyzed was relatively small, but we found that elutriation was effective at enriching autologous PBMC products for lymphocytes and depleting the product of monocytes and granulocytes.

When compared to methods that we have previous used to enrich PBMC concentrates for T cells or lymphocytes, anti-CD3/CD28 bead selection and bead selection plus plastic adherence, beginning manufacturing with elutriated lymphocytes resulted in greater T cell yields. Furthermore, the yield of T cells transduced with GD2-CAR was greatest when manufacturing was initiated with elutriated lymphocytes, but this was not the case with CD19-CAR T cell products. While the overall T cell yields for CD19-CAR T cell products was greatest when manufacturing was initiated with elutriated lymphocytes, the CD19-CAR T cell transduction efficiency was less for the cells produced from elutriated lymphocytes and there was no significant increase in the yield of transduced CD19 CAR T cells.

Among the 13 CD19- and GD2-CAR T cells products manufactured using elutriated lymphocytes, all products contained enough transduced T cells to meet protocol dose requirements. We have previously found that CAR T cells manufactured using T cells selected with anti-CD3/CD28 beads failed to contain sufficient quantities of transduced CD3+ cells to meet protocol dose criteria in 4 of 28 CD19-CAR T cell patients and none of 6 GD2-CAR T cell patients [11]. A change in the manufacturing process to include a monocyte depletion step using plastic adherence reduced the number of products that did not meet dose criteria to 1 of 20 patients; none of 15 CD19-CAR T cell products and to 1 of 5 GD2-CAR T cell products [11].

The finding that beginning manufacturing with elutriated lymphocytes improved GD2-CAR T cell expansion more than CD19-CAR T cells is likely due to the presence of greater quantities of myeloid cells in autologous PBMC concentrates used to manufacture GD2-CAR T cells. Most patients treated with GD2-CAR T cells had osteosacrcoma who are known to have circulating myeloid-derived suppressor cells (MDSCs) [14]. The patients treated in this study with CD19-CAR T cells most often had ALL and patients with hematologic malignancies are less likely to have circulating MDSCs [15, 16].

One limitation of the use of elutriation to isolate lymphocytes for CAR T production is that the elutriated lymphocyte fraction not only contains T cells, but it also contains B cells and NK cells and RBCs. A portion of acute lymphocyte leukemia blast cells are also found in the lymphocyte fraction. While we continue to initiate GD2-CAR T cell manufacturing with elutriated lymphocytes, we are investigating other, more specific methods of T cell purification for manufacturing CAR T cells for patients with ALL in order to improve T cell expansion and to exclude leukemic cells from being transduced.

## Conclusions

We found that counter flow elutriation of PBMC concentrates collected from children and young adults with ALL and sarcoma produced lymphocyte fractions with reduced quantities of neutrophils and monocytes. CAR T cells can be consistently produced and expanded from elutriated lymphocytes. These results provide further evidence of the importance of beginning CAR T cell manufacturing with lymphocytes that contain few contaminating myeloid cells.

## Abbreviations

ALL: acute lymphocytic leukemia
BCMA: B cell maturation antigen
CAR: chimeric antigen receptors
MDSC: myeloid-derived suppressor cells
NCI: National Cancer Institute
PBMC: peripheral blood mononuclear cells
RBCs: red blood cells
